# Phosphatidylcholine-Derived Lipid Mediators: The Crosstalk Between Cancer Cells and Immune Cells

**DOI:** 10.3389/fimmu.2022.768606

**Published:** 2022-02-15

**Authors:** Renata de Freitas Saito, Luciana Nogueira de Sousa Andrade, Silvina Odete Bustos, Roger Chammas

**Affiliations:** Centro de Investigação Translacional em Oncologia (LIM24), Departamento de Radiologia e Oncologia, Faculdade de Medicina da Universidade de São Paulo and Instituto do Câncer do Estado de São Paulo, São Paulo, Brazil

**Keywords:** lipid metabolism, phosphatidylcholine, lipid mediators, immunoregulation, immune microenvironment, cancer drug resistance

## Abstract

To become resistant, cancer cells need to activate and maintain molecular defense mechanisms that depend on an energy trade-off between resistance and essential functions. Metabolic reprogramming has been shown to fuel cell growth and contribute to cancer drug resistance. Recently, changes in lipid metabolism have emerged as an important driver of resistance to anticancer agents. In this review, we highlight the role of choline metabolism with a focus on the phosphatidylcholine cycle in the regulation of resistance to therapy. We analyze the contribution of phosphatidylcholine and its metabolites to intracellular processes of cancer cells, both as the major cell membrane constituents and source of energy. We further extended our discussion about the role of phosphatidylcholine-derived lipid mediators in cellular communication between cancer and immune cells within the tumor microenvironment, as well as their pivotal role in the immune regulation of therapeutic failure. Changes in phosphatidylcholine metabolism are part of an adaptive program activated in response to stress conditions that contribute to cancer therapy resistance and open therapeutic opportunities for treating drug-resistant cancers.

## Introduction

Cancer cells are characterized by their eximious ability to adapt and survive within harsh microenvironments (poor oxygenation and nutrient deprivation). Cancer metabolic plasticity is among the adaptive responses that allow tumor development in these conditions and also contribute to therapy resistance. The first tumor metabolic adaptation was identified by Otto Warburg in the 1920s, who described that cancer cells have an exacerbated glucose uptake and glycolysis accompanied by increased lactate production even under aerobic conditions (1). Since this pioneering work, known as the “Warburg effect”, much effort has been made to exploit the unique features of tumor metabolic phenotypes and metabolic reprogramming that is currently well-recognized as one of the hallmarks of cancer (2, 3). In recent years, lipid metabolism reprogramming has received renewed interest in the cancer field, and compelling evidence reveals the contribution of lipid remodeling in regulating the hallmarks of cancer (4).

Uncontrolled cell division exhibited by cancer cells introduces a cellular metabolic challenge, since it is necessary to double the total biomass (nucleic acid, proteins, and lipids) to support the mitotic cell division of a single cell into two equal-sized daughter cells. Cancer cells reprogram their metabolism from catabolism to anabolism to attend to this energetic and biomass demand to fuel cell proliferation (5). Among the biomolecules that compose total cell biomass, lipids have received fewer research efforts mainly due to their extremely diverse structure that turns their detection and quantification an analytical challenge. However, this scenario has changed due to technological progress in analytical approaches for lipid investigation that helped to gain a comprehensive look at the complexity and singularity of tumor lipid metabolism (6). Advances in two main analytical techniques, magnetic resonance spectroscopy (MRS) and mass spectrometry (MS) often coupled to liquid chromatography (LC) systems, contributed to the identification of abnormal choline (Cho) metabolism in tumors. Over the past four decades, accumulating evidence of MRS studies evaluating total choline (tCho) metabolite levels in cancer cells, notably free choline (Cho), phosphocholine (PCho), and glycerophosphocholine (GPC), revealed the importance of choline metabolism in tumor biology. Almost every tumor cell type investigated showed increased levels of tCho metabolites compared to non-malignant counterparts (7–15).

**Figure 1:**
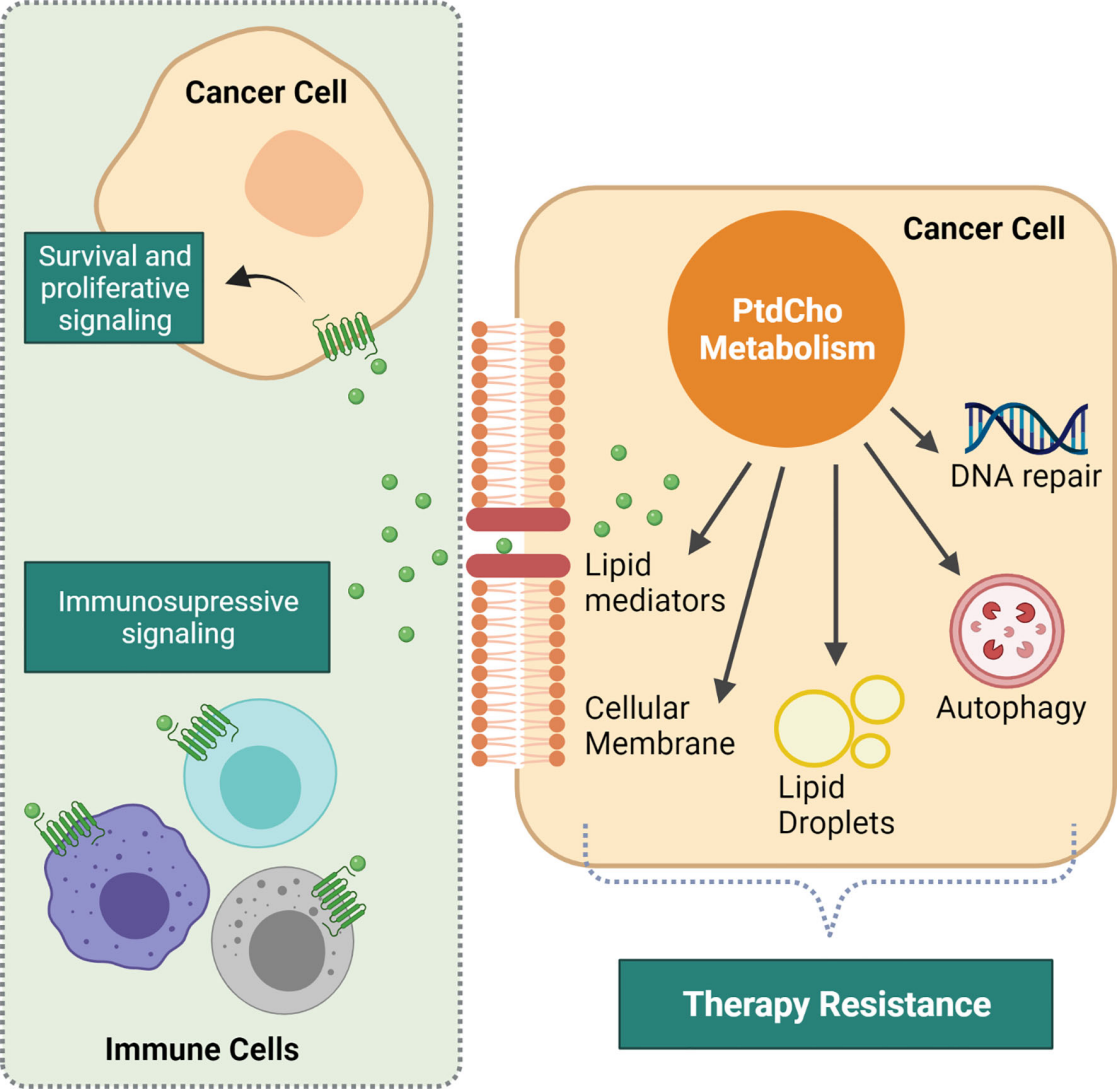
Intracellular and intercellular consequences of altered phosphatidylcholine (PtdCho) metabolism that impacts response to therapy. Increased PtdCho metabolism supports cancer cell accelerated growth by providing the major cellular membrane component. Additionally, PtdCho promotes intracellular events that mediate resistance to therapy, such as DNA repair, lipid droplet synthesis, and autophagy process. PtdCho-derived lipid mediators are prominent drivers of resistance. They are recognized by their cognate receptors present both in cancer cells and immune microenvironment cells, driving cancer cell survival and proliferation and promoting immunosuppression. Created with BioRender.com.

Cho-containing phospholipids are the most abundant phospholipids in eukaryotic cell membranes, and phosphatidylcholine (PtdCho) is the predominant phospholipid (<50%) in most mammalian membranes. Notably, cancer cells accumulate Cho-containing metabolites that are precursors or breakdown products of PtdCho to fuel their anabolic phenotype with building blocks and to promote intracellular processes that contribute to drug resistance. Additionally, hydrolysis of PtdCho generates lipid mediators that exert an intercellular crosstalk favoring cancer cell survival, proliferation, and immune modulation that culminate in resistance to therapy (**Figure 1**). Here, we highlight the role of PtdCho as a molecular link between altered choline metabolism and cancer therapy resistance. We start defining PtdCho-mediated protumoral signaling in a cancer cell perspective and further extend our discussion on the immune modulation of PtdCho-derived lipid mediators. In addition, we list some studied therapeutic strategies to intervene in the PtdCho metabolism and emphasize the importance to increase the knowledge of this lipid metabolism due to the complexity of the intracellular and intercellular signaling of PtdCho-mediated resistance to therapy.

## Phosphatidylcholine Metabolism and Cancer

PtdCho is a glycerophospholipid consisting of a choline headgroup and a phosphate group substituent linked to two fatty acid chains (**Figure 1**). Of note, choline is an essential nutrient obtained from dietary sources or by degradation of choline-containing lipids, and once inside the cell, the main fate of choline is PtdCho synthesis. Considering that cancer cells exhibit elevated levels of choline-containing lipids, it is appropriate to assume that cancer cells have efficient lipidic feedback to sustain an elevated choline metabolism.

**Figure 2:**
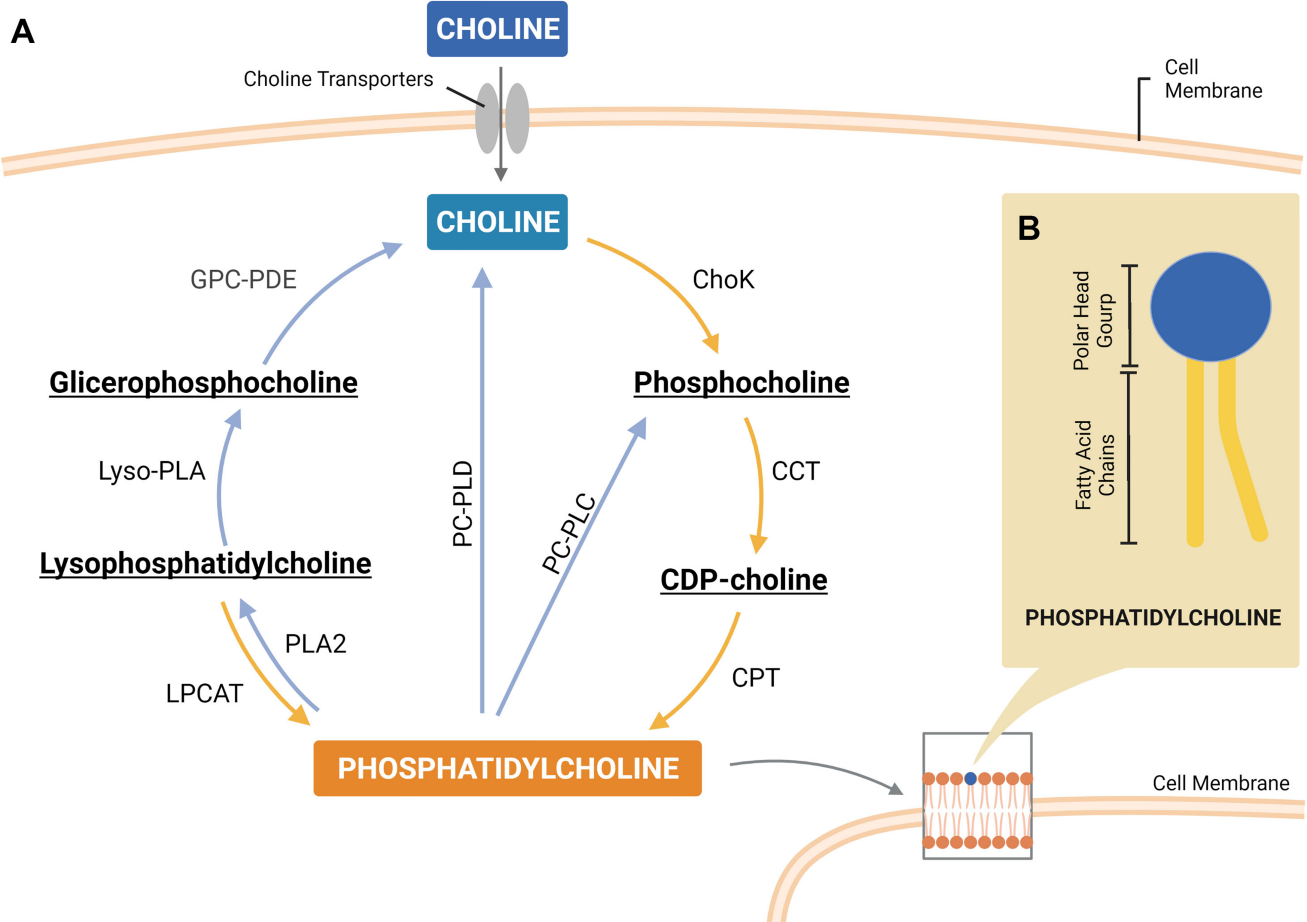
A simplified scheme of the phosphatidylcholine (PtdCho) cycle, highlighting the enzymes involved in PtdCho synthesis (Kennedy pathway) and catabolism **(A)**. Representative structure of PtdCho consisted of a choline head group and a phosphate group (polar head group) linked to two fatty acid chains by a glycerol moiety **(B)**. ChoK, choline kinase; CCT, CTP:phosphocholine cytidylyltransferase; CPT, CDP-choline cholinetransferase; LPCAT, lysophosphatidylcholine acyltransferase; PLA2, phospholipase A2; PC-PLC, phosphatidylcholine-specific phospholipase C; PC-PLD, phosphatidylcholine-specific phospholipase D; lyso-PLA, lysophospholipase; GPC-PDE, glycerophosphocholine phosphodiesterase. Created with BioRender.com.

To understand how and why cancer cells accumulate choline metabolites, either PtdCho precursors or products, we start summarizing the biosynthetic pathway of this lipid. Over 65 years ago, Eugene Kennedy elucidated the *de novo* biosynthetic pathway of PtdCho, known as Kennedy pathway or CDP-choline pathway (**Figure 2**) (16). PtdCho is predominantly synthesized through the CDP-choline pathway in all mammalian cells with choline as the first substrate of a sequential cascade of enzymatic alterations that result in PtdCho formation. In this pathway, choline obtained from an external medium or available in the cytosol by the breakdown of choline-containing compounds is phosphorylated by choline kinase (ChoK). In the rate-limiting second step, phosphocholine (PCho) is converted into the high‐energy intermediate CDP-choline by the enzyme CTP: phosphocholine cytidylyltransferase (CCT). Subsequently, the enzyme CDP-choline cholinetransferase (CPT) catalyzes the final reaction using CDP-choline and diacylglycerol (DAG) to form PtdCho (17).

After the final step of PtdCho synthesis, which occurs in the endoplasmic reticulum (ER) membrane, this phospholipid is transported and delivered to other organelles, such as cell membrane, by different inter-organelle mechanisms of transport (18). PtdCho is asymmetrically distributed across the lipid bilayer membrane and is enriched in the outer leaflet, comprising 40%–50% of total phospholipids. PtdCho also serves as a precursor of two other major membrane phospholipids, sphingomyelin (SM) and phosphatidylethanolamine (PtdEth). Thus, PtdCho has a crucial role as a direct or indirect source of structural building blocks for cellular membranes. However, PtdCho is more than a structural component of mammalian membranes, it is also an important source of lipid second messengers. PtdCho catabolism generates signaling molecules such as phosphatidic acid (PA), DAG, lyso-PC, and arachidonic acid (AA) that have protumoral effects. Additionally, degradation of PtdCho releases choline for replenishment of intermediates in the CDP-choline pathway. It is reasonable to postulate that the PtdCho cycle of synthesis and catabolism (**Figure 2**) supports the maintenance of the proliferative phenotype of cancer cells and contributes to protumoral characteristics that allow tumor progression and culminate in resistance to therapy.

## The Molecular Origins of Deregulated Choline Metabolism in Cancer

The role of increased levels of Cho metabolites was initially interpreted as a means to meet the demands of fast-growing cancer cells. Indeed, increased consumption of choline and secretion of PtdCho by cancer cells are positively correlated with cell proliferation rates (19). However, Daly et al. (20) demonstrated *in vitro* that proliferative non-malignant cells maintain lower PCho and tCho levels compared to cancer cells (20), revealing that altered choline metabolism is not only supportive to cell proliferation but is also linked to malignant transformation and cancer progression (11). This assertion is supported by *in vitro* studies showing that both tCho and PCho levels increase in the malignant transformation of human mammary (21) and prostate (7) epithelial cells. Additionally, PCho accumulation is also associated with a more aggressive cancer phenotype (7, 21–23). The knowledge about the molecular mechanisms that regulate choline metabolism in cancer is growing, and we underline some of these mechanistic insights.

### Increased Choline Uptake by Cancer Cells

Considering that fatty acids are substrates to PtdCho synthesis, the increased ratio of PtdCho biosynthesis in cancer can be in part a response to the enhanced fatty acid synthesis frequently observed in cancer cells (24). Additionally, it is intuitive thinking that one of the causes of cholinic phenotype is an enhanced ability of importing free extracellular choline by tumor cells. Of note, choline does not cross cell membranes by passive diffusion, being dependent on a choline transport system composed of four transporter families categorized according to their affinity for choline, high-affinity choline transporter 1, choline transporter-like proteins, polyspecific organic cation transporters, and organic cation/carnitine transporters. Several studies have underlined increased expression of each subtype of choline transporter in different human cancer cell lines in comparison with normal counterparts (11, 25). Given the fact that choline uptake is a rate‐limiting step in phospholipid metabolism and a prerequisite for cancer cell proliferation, the inhibition of choline transporters in cancer cells results in lower levels of intracellular choline accompanied by cell death induction (26, 27). As a consequence of decreased intracellular choline levels, there is also a reduction of PtdCho and PCho levels. In this context, cancer cells hydrolyze sphingomyelin as a compensatory response to maintain the generation of PtdCho and PCho. However, sphingomyelinase‐catalyzed hydrolysis of sphingomyelin also generates apoptosis-inducing factor ceramide, which activates caspase-3 and results in apoptotic cell death induction (28). Notably, the increased capacity of the cancer cell to import extracellular choline is a major contributor to the cholinic phenotype. However, further molecular characterization is needed to define what orchestrates the different combinations of choline transporters and how they lead to enhanced choline transport in cancer cells to drive the discovery of potential cancer targets.

### Enhanced Activity of Choline Metabolic Enzymes Mediated by Oncogenic Regulation

The PtdCho cycle is composed of a network of enzymes whose expression and activity can be modulated by genetic alterations present in cancer cells. Increased intracellular levels of PCho in cancer cells are mainly attributed to upregulation of ChoK enzyme and also partially derived from phosphatidylcholine-specific phospholipase C (PC-PLC) and phosphatidylcholine-specific phospholipase D (PC-PLD) enhanced activity.

Accordingly, the ChoKα isoform, which catalyzes the phosphorylation of Cho to PCho, is upregulated in epithelial ovarian (13), breast (25), bladder (29), and colon (22) cancer cells. In addition to this metabolic function, *in vitro* and *in vivo* pieces of evidence show that ChoKα overexpression contributes to tumor progression, metastasis, and aggressiveness (29). Moreover, ChoKα overexpression has a prognostic significance and predicts poor prognosis of colorectal cancer (30), early-stage non-small cell lung cancer (31), and hepatocellular carcinoma patients (32). Importantly, PCho can also be generated by the breakdown of PtdCho through PC-PLC activity. PC-PLC is upregulated in ovarian and breast cancer cells of different subtypes and accounts for 20%–50% of intracellular PCho production (15, 33). Moreover, *in vitro* inhibition of PC-PLC activity resulted in cell proliferation arrest in both cancer models (13, 34). In particular, a decrease of migration and invasion potential, together with a loss of mesenchymal traits, was observed after treatment of breast cancer cells with a PC-PLC inhibitor (15). Additionally, PC-PLD hydrolyzes PtdCho in PA and free choline, which can reenter the CDP pathway and generate choline intermediates. PC-PLD expression is elevated in diverse cancer types, such as gastric (35), breast (36, 37), epithelial ovarian (13), and melanoma (38). Evidence shows that PC-PLD regulates multiple tumor cell events, such as cell transformation, proliferation, survival, and migration (39).

Mechanistic investigation revealed that PtdCho metabolic enzyme activation is dependent on oncogenic signaling pathways, mainly the oncogene *ras* that affects the activities of ChoK, PC-PLC, and PC-PLD enzymes. Glunde et al. (11) describe the oncogenic signaling pathways involved in the regulation of choline metabolism enzymes (11). In this sense, *ras*-transformed cells exhibit increased ChoK activity accompanied by increased levels of its product, PtdCho (40–42). Further investigation revealed that in mammalian cells, the mechanism of ChoK regulation by *ras* implies the involvement of two *ras* effectors, RAL GTPase guanine nucleotide dissociation stimulator (RALGDS) and Phosphoinositide 3-kinase (PI3K) signaling (41). Several studies underline that the oncogene *ras* also regulates the activity of PC-PLD enzyme (39, 43, 44). Moreover, the enzyme PC-PLC is a downstream target of Ras, and its activation plays an important role in inducing Ras-mediated mitogenic signaling (45, 46). Thus, oncogene-driven activation of ChoK, PC-PLD, and PC-PLC enzymes increases PtdCho synthesis and degradation, leading to the accumulation of energy-rich molecules and providing sources for cancer cell proliferation.

## The Consequences of Deregulated Choline Metabolism in Cancer

### Phosphatidylcholine Accumulation Confers Metabolic Flexibility to Cancer Cells’ Survival Under Stress Conditions

Cancer choline metabolism is also modulated by harsh tumor microenvironment (TME) conditions, mainly hypoxic and nutritional stress. A large number of studies indicate increased levels of tCho-containing compounds in cancer cells (47). It is interesting to stress out that these choline metabolites were observed to be heterogeneously distributed in tumor sections (48–50). This is of particular interest, as Glunde et al. (51) demonstrated *in vivo* that hypoxic areas of human prostate tumor xenografts contain increased tCho levels (51). Additionally, they reported that *in vitro* exposure of prostate cancer cells to hypoxia generates increased levels of PCho and tCho as well as increased expression of ChoK. They also provided mechanistic insights into how hypoxia induces choline metabolism by demonstrating that Hypoxia Inducible Factor-1, HIF-1 directly binds to *ChoKα* promoter region. Similarly, intermittent hypoxia also upregulates *ChoK* in rat pheochromocytoma PC-12 cells (52). In contrast, opposing evidence shows a decrease in choline levels in cancer cells exposed to hypoxic conditions without loss of cell viability (53). A hypoxia-mediated inhibition of choline phosphorylation has also been demonstrated in cancer cells (53, 54) as a result of a decrease in ChoK expression and activity mediated by HIF-1α (55).

**Figure 3:**
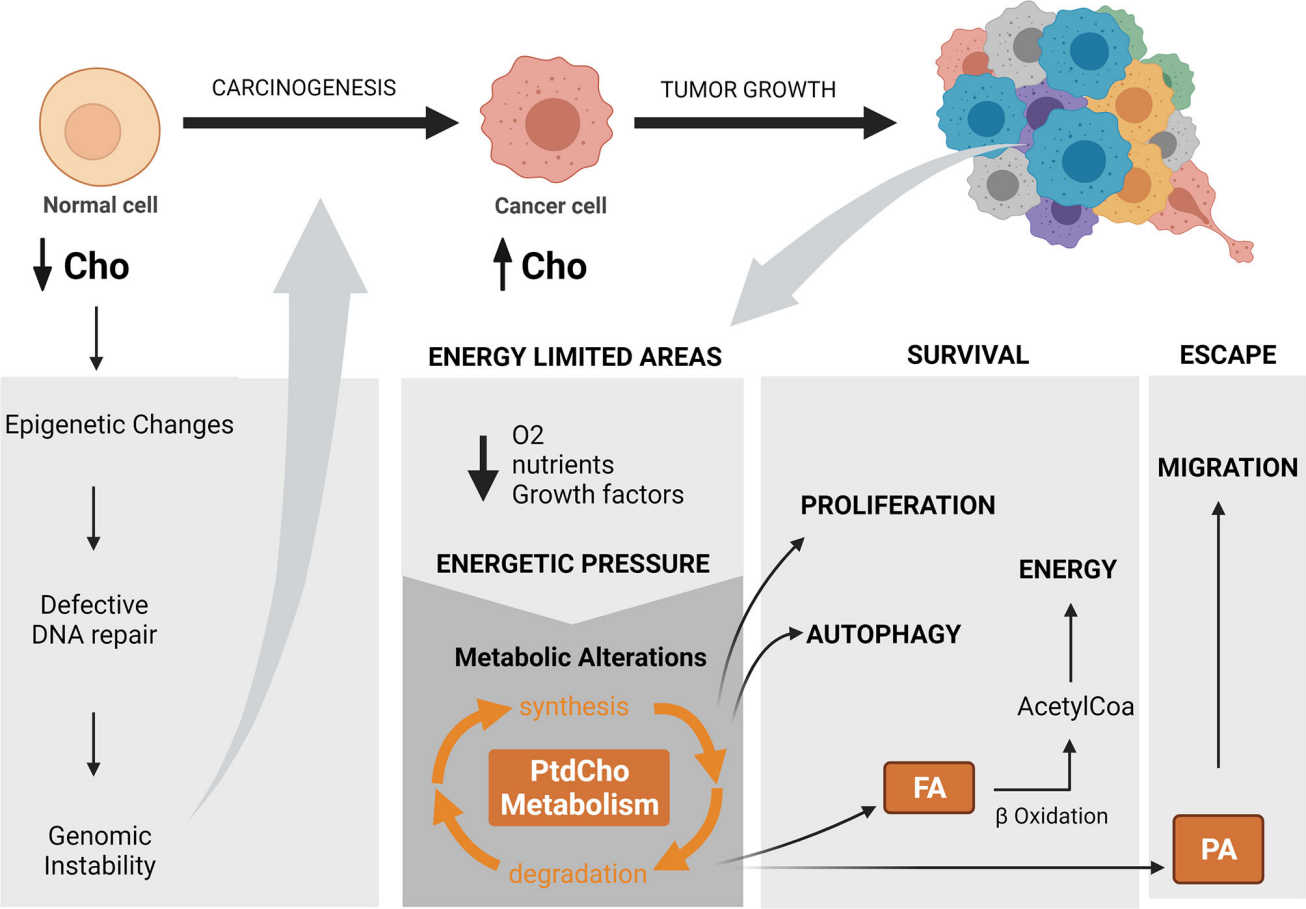
The impact of altered choline metabolism in carcinogenesis and tumor progression. Altered choline metabolism promotes carcinogenesis through modulating DNA repair gene expression by methylation and generating genomic instability. Energy-limited areas arise with tumor growth and exert an energetic pressure that leads to metabolic reprogramming and results in diverse metabolic phenotypes. Altered choline metabolism is among these phenotypes. In particular, phosphatidylcholine metabolism regulates adaptative cellular processes, such as proliferation and autophagy that allow cancer cell survival with limited energetic sources and in parallel induce migration as an escape route toward an energy-privileged area. Cho, choline; PtdCho, phosphatidylcholine; FA, fatty acid; PA, phosphatidic acid; O_2_, oxygen.Created with BioRender.com.

Importantly, Glunde et al. (51) observed that not all tumor areas with high tCho levels were colocalized with hypoxic regions, indicating that other tumor environment conditions can also modulate choline metabolism. Acidosis can elicit opposite effects in PtdCho synthesis once *in vitro* evidence shows that it inhibits ChoK but enhances CCT (CTP:phosphocholine cytidylyltransferase) enzyme activity with a net increase of PtdCho pool in low pH conditions (56). However, another *in vitro* evidence shows that acidosis decreases PCho levels, reinforcing that ChoK is inhibited by low pH, but also increases GPC levels, indicating enhanced PtdCho degradation (57). Of note, GPC production from PtdCho catabolism is mediated by lysophospholipase, and phospholipase A2 catalyzed reactions with the generation of free fatty acids. It is interesting to note that acidosis can inhibit glycolysis in human cancers (58) and, as a consequence, result not only in a reduction of ATP generation but also in decreased amounts of acetyl-CoA, which feeds the tricarboxylic acid (TCA) cycle for aerobic respiration. Thus, these pieces of evidence drive us to suggest that during acidosis, decreased PtdCho synthesis and increased PtdCho breakdown allow cancer cells to fuel beta-oxidation of fatty acids as a source of acetyl-CoA (**Figure 3**).

In this sense, ample evidence supports the notion that in aerobic conditions, cancer cells exhibit increased choline uptake to activate anabolic metabolic pathways to sustain their high proliferative rates. On the contrary, hypoxic conditions have been reported to diminish choline uptake while enhancing glucose and acetate in cancer cells (53). Kamphorst et al. (59) demonstrated that during hypoxia, acetate is the major additional source of carbon donor for acetyl-CoA, and the generation of this precursor for fatty acid biosynthesis allows cancer cells to maintain lipogenesis and proliferation under hypoxic conditions (59). Interestingly, the *in vitro* study by Yoshimoto et al. (60) revealed that the rate of acetate incorporation in tumor cells under hypoxia is superior to the rate observed in normal cells, and the metabolic fate of acetate in hypoxic cells was preferentially incorporated into PtdCho (60). Importantly, they also showed that this increased acetate uptake and lipid incorporation were positively correlated with growth activity. Thus, cancer cells might have these metabolic adaptations to maintain the anabolism of fatty acids, which require acetyl-CoA units to compensate for inhibition of glycolytic ATP and acetyl-CoA production by activation of fatty acids as a source of energy.

### Phosphatidylcholine Metabolism Promotes a Tumor Escape Route From Energetic Stress

There is a correlation between survival and cancer cell migration under stress conditions, suggesting that cancer cells in addition to surviving and suppressing cell death also trigger a migration phenotype to escape from stressful regions (61). This raises the question of how migration is triggered by stressful conditions and if enhanced choline metabolism could be a linker of these phenomena.

Oxygen- and nutrient-deprived areas arise as a consequence of inadequate blood supplies during solid tumor growth and impose an energetic pressure on cancer cells. To survive, cancer cells need to first suppress cell death induced by these stress conditions and ultimately provide means for obtaining energy. One possibility is that cancer cells with increased amounts of choline compounds have the advantage of obtaining energy and building blocks from the degradation of these lipids. Alternatively, cancer cells in parallel induce migration to sites where nutrition could be found, and choline compound storage can contribute to this process by their breakdown products. In this sense, Zheng et al. (62) revealed that under stress caused by serum withdrawal, MDA-MB-231 human breast cancer cells exhibited increased PC-PLD enzyme activity concomitant to an enhanced migration and invasion potential (62). Compelling evidence gained from PLD2 overexpression (63), an isoform of *PC-PLD* gene, in low-invasive breast cancer cells resulted in the conversion of these cells into a highly aggressive phenotype with increased capacity of lung metastasis formation, which was inhibited by two different small-molecule inhibitors of PC-PLD activity (63). Animals deficient in another *PC-PLD* gene isoform, *PLD1*, or treated with a small-molecule inhibitor of PLD1 activity, exhibited reduced tumor growth, angiogenesis, and metastasis (63). Aberrant expression of both PC-PLD isoforms has been detected in different cancers and linked to cancer cell survival and a pro-metastatic phenotype through activation of different signaling pathways revised in Yao et al. (64). Thus, a stressful tumor environment can drive PtdCho degradation, in particular through PC-PLD hydrolysis, which contributes to a cancer cell migration program. These data indicate PC-PLD as a potential target for cancer therapy and point toward a small-molecule dual inhibitor of PLD1 and PLD2 as a promising strategy.

### Phosphatidylcholine and Phosphatidylcholine-Derived Lipid Mediators Regulate Cancer Cell Growth and Survival

PtdCho metabolism has been linked to opposing cellular events, cell proliferation, and cell death. Noticeably, the cell cycle controls PtdCho homeostasis to avoid an excess or deficit of membranes. Essentially, PtdCho metabolism is modulated during the cell cycle and is characterized by a high rate of PtdCho degradation and resynthesis in the G1 phase, a reduced PtdCho turnover that leads to doubling PtdCho amounts in the S phase, and a cessation of PtdCho metabolism in G2/M phases (65). In the opposite direction, the first evidence of a direct link between cell death and PtdCho synthesis was a report showing that Chinese hamster ovary (CHO) cells with a mutation in the CCT enzyme resulted in PtdCho depletion and concomitant apoptosis induction (66). The molecular mechanism by which PtdCho depletion is sensed and transduced to cell death has not yet been fully elucidated; however, evidence shows that inhibition of PtdCho synthesis triggers apoptosis through a mechanism that involves the activation of an endoplasmic reticulum, ER stress response (67, 68). Moreover, PtdCho is a substrate for sphingomyelin (SM) synthesis, and the final step of this biosynthetic route involves the exchange of the phosphocholine head group from PtdCho to ceramide. Yen et al. (69) demonstrated that in parallel to the intracellular decrease of PtdCho, SM levels also decrease and the apoptosis inducer ceramide accumulates (69).

More recently, studies have implicated lipid metabolism in the non-apoptotic cell death process of ferroptosis. This process is characterized by the accumulation of iron-dependent lethal lipid peroxides (LPOs) that can be generated from the oxidation of phospholipids, such as arachidonoyl and adrenoyl, by the catalysis of acyl-CoA synthetase long-chain family member 4 (ACSL4), LPCAT3, and 15-lipoxygenase (15-LOX/ALOX15) (70, 71). A detailed underlying mechanism of ferroptosis in cancer biology was reviewed by Li and Li (72). Chemotherapy and mainly ionizing radiation (IR) therapy generate reactive oxygen species (ROS) that can target lipid peroxidation and cause ferroptosis induction. Of interest, IR was reported to induce ferroptosis, and inhibition of ACSL4 enzyme activity reverted IR-induced ferroptosis and promoted radioresistance (73, 74). While oxidative metabolites from arachidonoyl and adrenoyl can generate "find me signals" and elicits an antitumor response, ferroptotic cancer cells have increased *PTGS2* gene expression, which encodes cyclooxygenase 2 (COX-2) to produce prostaglandin E2 (PGE2), a major pro-inflammatory factor (75). These data suggest that ferroptosis and lipid metabolism may be involved in resistance to therapy. Further research to expand the understanding of the unique features of ferroptosis will unveil the therapeutic windows to precisely target this process.

Importantly, PtdCho depletion can indirectly interfere in cell viability once it is an important source of lipid mediators that are known to regulate cell growth, such as PA and DAG. Accordingly, a balance between mitogenic and antimitogenic lipid mediators derived from PtdCho can dictate the fate of cells toward cell proliferation, arrest, or death (76). This is of particular interest in oncology once several antitumoral drugs, including chemotherapy and radiotherapy, induce an increase in proapoptotic ceramide levels and parallel mitogenic DAG levels (77).

## The Impact of Cancer Therapy Response on Phosphatidylcholine Metabolism

Since the cholinic phenotype, characterized by elevated PCho and high tCho-containing metabolites, is considered a metabolic hallmark of cancer (11, 21), some groups started to explore the use of choline metabolite biomarkers to monitor tumor response (78). Several reports have shown that choline-containing metabolites are modulated by antitumoral therapy (79–83). Nishio et al. (81) showed that PtdCho levels were decreased by 50% in human lung adenocarcinoma cells treated with cisplatin *in vitro* (81). Additionally, a decrease in PCho levels and an increase in GPC levels were observed in breast cancer cells *in vitro* in response to doxorubicin (79) and *in vivo* after docetaxel treatment (84). In contrast, chemotherapy was also reported to increase choline metabolites. Notably, PtdCho levels were increased in breast cancer cells by doxorubicin treatment (79) and in human colon cancer cells and tumor xenografts by histone deacetylase (HDAC) inhibitors (85). Additionally, increased levels of CDP-choline were observed in human promyelocytic leukemia (HL-60) and Chinese hamster ovary (CHO-K1) after treatment with several cytotoxic drugs (86). PCho levels were also increased in human neutrophils undergoing apoptosis (87). Overall, these divergent data indicate that changes in choline metabolism can be treatment-specific and cellular context-dependent.

Concerning enzymatic inhibitors, in pediatric glioblastoma, PCho, tCho, and choline kinase alpha (ChoKα) protein levels decreased upon PI3K pathway inhibition, whereas an increase in PCho, glycerophosphocholine (GPC), and tCho was observed in response to temozolomide (TMZ). Since these metabolic changes can be monitored by non-invasive techniques like NMR, the authors suggested that monitoring Cho metabolism might represent a potential biomarker for monitoring response in pediatric gliomas (88). Furthermore, choline and PCho metabolism can also be altered in response to certain treatments such as histone HDAC, phospholipase Cγ1, Mitogen Activated Protein Kinase, MAPK, PI3K, and Heat Shock Protein 90, HSP90 inhibitors (89–95). Regarding the use of HDAC, which is approved for cutaneous T-cell lymphoma treatment, Beloueche-Babari et al. (85) showed that HDAC inhibition led to an increase in *de novo* phosphocholine synthesis that was accompanied by ChoKα expression in colon and prostate carcinoma cells *in vitro* and *in vivo* (85). This modification in choline metabolism is also observed in response to radiotherapy. In xenograft pancreatic tumors, an increase in choline and a decrease in glycerophosphocholine + phosphocholine in comparison to the normal pancreas was reported by a study in 2013 (96). Moreover, the authors observed that, in response to different doses of radiotherapy, choline levels were diminished and glycerophosphocholine + phosphocholine increased.

Although there are apparent discrepancies about the increase or decrease in some choline-containing metabolites, ^1^H-MRS imaging of tCho levels in many cancers has been used to improve treatment monitoring and therapy strategy, as also proposed by Katz-Brull et al. (97), Mignion et al. (98), and Al-Saffar et al. (99) (97–99). In a retrospective study, patients with locally advanced breast cancer that responded or did not to neoadjuvant chemotherapy were differentiated by a reduction in tCho levels (100). In line with this finding, Meisamy et al. (78) reported a reduction in PCho levels as early as 24 h after the first treatment in locally advanced breast cancer patients who responded to doxorubicin chemotherapy, while it remained the same or increased in non-responders (78). An early response to therapy associated with a reduction of tCho was also observed in prostate cancer (101, 102). In contrast, a transient increase in choline compounds was observed in Breast Cancer gene 1, BRCA-1 mouse mammary tumors sensitive to docetaxel treatment (103). These results imply that quantitative changes in tCho levels could be a parameter to predict early tumor response, which would be valuable to guide the clinician in determining an alteration in the dosage of the drug and administration of alternative drugs or offer surgery options to non-responders.

Considering that PCho concentration correlates strongly with cell proliferation (104), one hypothesis is that a decrease in choline metabolites after therapy may reflect cell cycle arrest. However, the molecular basis of how chemotherapy interferes in choline metabolism has been investigated to clarify the molecular mechanisms behind this effect. Accordingly, a cisplatin-induced decrease in PtdCho levels was attributed to an increase in PC-PLC activity (81) and doxorubicin-induced decrease in PCho levels to downregulation of PLD1, ChoKα, and glycerophosphodiester phosphodiesterase domain containing 6 (GDPD6) enzymes (79). Thus, therapy-induced PCho and PtdCho increased levels could reflect an increase in *de novo* synthesis through ChoK activity and/or a decrease in the degradative pathways mediated by PC-PLC or PC-PLD activity. The decrease in PCho levels observed posttreatment is frequently coupled with an increase in GPC levels. Considering that PCho is an anabolite and GPC a catabolite of PtdCho, a decrease in PCho/GPC ratio after treatment implies a net increase in PtdCho turnover. In line with this, evidence shows that HDAC inhibitors result in a net augmentation of PtdCho by positively modulating the expression of CTP-PC cytidylyltransferase, the rate-limiting enzyme in PtdCho biosynthesis together with the observation that PtdCho breakdown product GPC is decreased after HDAC inhibitor treatment (85, 105). Interestingly, the augmentation of PtdCho was not translated to increases in cell volume, suggesting that it was not used to synthesize new outer membrane. In line with this, the accumulation of PtdCho can be an important reservoir of PtdCho-derived lipid mediators that can drive cancer survival and resistance to therapy.

## Changes in Phosphatidylcholine Metabolism Contribute to Drug Resistance

Treatment failure in cancer patients is closely related to the development of drug resistance. Thus, it is crucial to elucidate the molecular processes that lead to drug resistance to intervene in these events and improve patient response to therapy. Few hints about lipid remodeling involvement in tumor resistance were reported several years ago. Back in the 1970s, Schlager and Ohanian (106), using guinea pig tumoral cells, observed that the metabolic inhibitors actinomycin D and Adriamycin were able to increase cell sensitivity to antibody-complement killing (106). Interestingly, this effect was accompanied by a reduction in PtdCho incorporation, among other lipids, into cellular organelles such as ER, nuclear membrane, mitochondria, and microsomes, suggesting that lipid synthesis might be involved in tumor resistance. The cellular mechanisms involved in acquired and intrinsic resistance are diverse and complex, and the understanding of how lipid metabolism modulates these pathways is still largely unknown. Overexpression of multidrug resistance (MDR) proteins is found in several tumor types and is associated with increased resistance to drug compounds due to the active efflux of a broad range of chemical molecules. In 1997, Bosch et al. (107) showed that PtdCho is a substrate for MDR1 P-glycoprotein (PgP) in T-cell leukemia resistant cells that might be responsible for the altered lipid composition between sensitive and resistant tumor cells as well as the inefficacy of treatments based on liposome delivery (107). In breast cancer resistant cells, tamoxifen, a broadly used agent in hormone therapy for estrogen-positive breast tumors, inhibited the uptake of choline probably by its action as an antagonist of PgP. Although the impact of this blockade had not been evaluated under these circumstances, the authors speculated that tamoxifen can interfere in choline metabolism (108). A study by Ramu et al. (109) revealed that the incorporation of choline in phosphocholine is decreased in drug-resistant leukemia cells in comparison to the parental cells (109). The authors also found that PtdCho synthesis could be restored through the inhibition of MDR inhibitors such as verapamil, indicating that sensitive and resistant tumor cells present different membrane lipid compositions that correlate to their sensitivity to a range of drugs used in cancer treatment impacting on the outcome. In 1992, Dubois and Tapiero (110) demonstrated an alteration in phospholipid metabolism characterized by an increase in PtdCho synthesis from PtdEth exclusively in leukemia resistant cells (110).

Some years later, the correlation between Cho/PtdCho, plasma membrane lipid composition, and drug sensitivity was demonstrated by others using different experimental approaches. Riedel et al. showed that proliferating pre-malignant Chang cells were more resistant to the FB1(fumonisin B1)-induced cytotoxicity compared to primary hepatocytes. Differences in lipid content, including lower PtdCho levels in Chang cells, which imply a more rigid plasma membrane, were partially responsible for this differential cell response to FB1. This finding reinforces the notion that lipid composition changes along with cell transformation and tumor progression, interfering in tumor response to cytotoxic therapy (111). In 2009, it was demonstrated that the upregulation of Cho transporter CHT1 and ChoK was involved in acquired resistance to chemotherapy in glioblastoma (GBM) (112). Concerning radiotherapy, Desoubzdanne et al. (113) compared choline metabolism between glioma radiosensitive and radioresistant cells (113). As reported by Vanpouille et al. (112), the authors found higher Cho and PCho levels and a global PtdCho metabolism more active in radioresistant cells. In this sense, NMR spectroscopy has also been used to investigate if changes in choline metabolism are associated with the MDR phenomenon. It has been demonstrated that choline metabolite spectra detected by ^31^P NMR are indeed different in resistant (drug-selected) cancer cells compared to drug-sensitive cells. In a model of MCF-7 human breast cancer cell induction of MDR with Adriamycin, a combined analysis of both ^1^H and ^31^P NMR spectra revealed that sensitive cells showed higher PCho concentrations than resistant cells, but choline levels were similar (114). In agreement, in an *in vivo* study with murine mammary adenocarcinoma, NMR revealed that Adriamycin-sensitive tumors have increased PCho and GPC levels compared to Adriamycin-resistant tumors (115). Additionally, in the same study, treatment of tumors with Adriamycin decreased PCho and GPC levels only in Adriamycin-sensitive tumors. On the contrary, another piece of evidence shows that docetaxel-sensitive tumors exhibited a lower level of choline compounds compared to their resistant counterparts (103). This inconsistency in choline metabolism change in MDR reinforces that these changes may depend strongly on the drug used for MDR induction and/or cancer cell type.

Albeit not universal, an increase in choline metabolites would likely be a predictive marker of drug resistance, and PtdCho metabolic enzymes are a linker of these phenomena. Evidence shows that breast cancer cells treated with doxorubicin increased PCho/GPC ratio caused by a downregulation of the enzymes PLD1, GDPD6, and ChoKα (**Figure 2**). Importantly, silencing of the metabolic enzymes PLD1 and ChoKα sensitized breast cancer cells to doxorubicin and specific GDPD6 silencing counteracted doxorubicin migration induction (79). Considering the role of ChoKα enzyme in the generation of PCho, high levels were consistently observed in cancer cells, and overexpression of this enzyme mediated an increase in MCF-7 breast cancer cell resistance to 5-fluorouracil together with a substantial increase in PCho level (116). Moreover, silencing of ChoKα enhanced the sensitivity of epithelial ovarian cancer to chemotherapeutic agents, such as platinum, doxorubicin, and paclitaxel (117). Importantly, the same sensitization effect of ChoKα silencing was observed in a drug-resistant context with platinum-resistant SKOV3 cell line (117). Following these findings, a study identified a group of super-enhancers (SEs) that are abnormally activated in castration-resistant prostate cancer resistant to enzalutamide antiandrogen drug. Among these SEs was the choline phosphotransferase 1 (*CHPT1*) gene, which encodes cholinephosphotransferase 1 (CPT) protein that catalyzes the last step of PtdCho synthesis (118). Indeed, *CHPT1* has been shown overexpressed in cancer and associated with tumor growth (119). Taken together, all these reports demonstrate that cancer therapy modulates the expression of PtdCho metabolic enzymes, which alter choline metabolite levels and render cancer cells resistant to treatment.

### Phosphatidylcholine as a Precursor of Lipid Mediators Involved in Therapy Resistance

**Figure 4:**
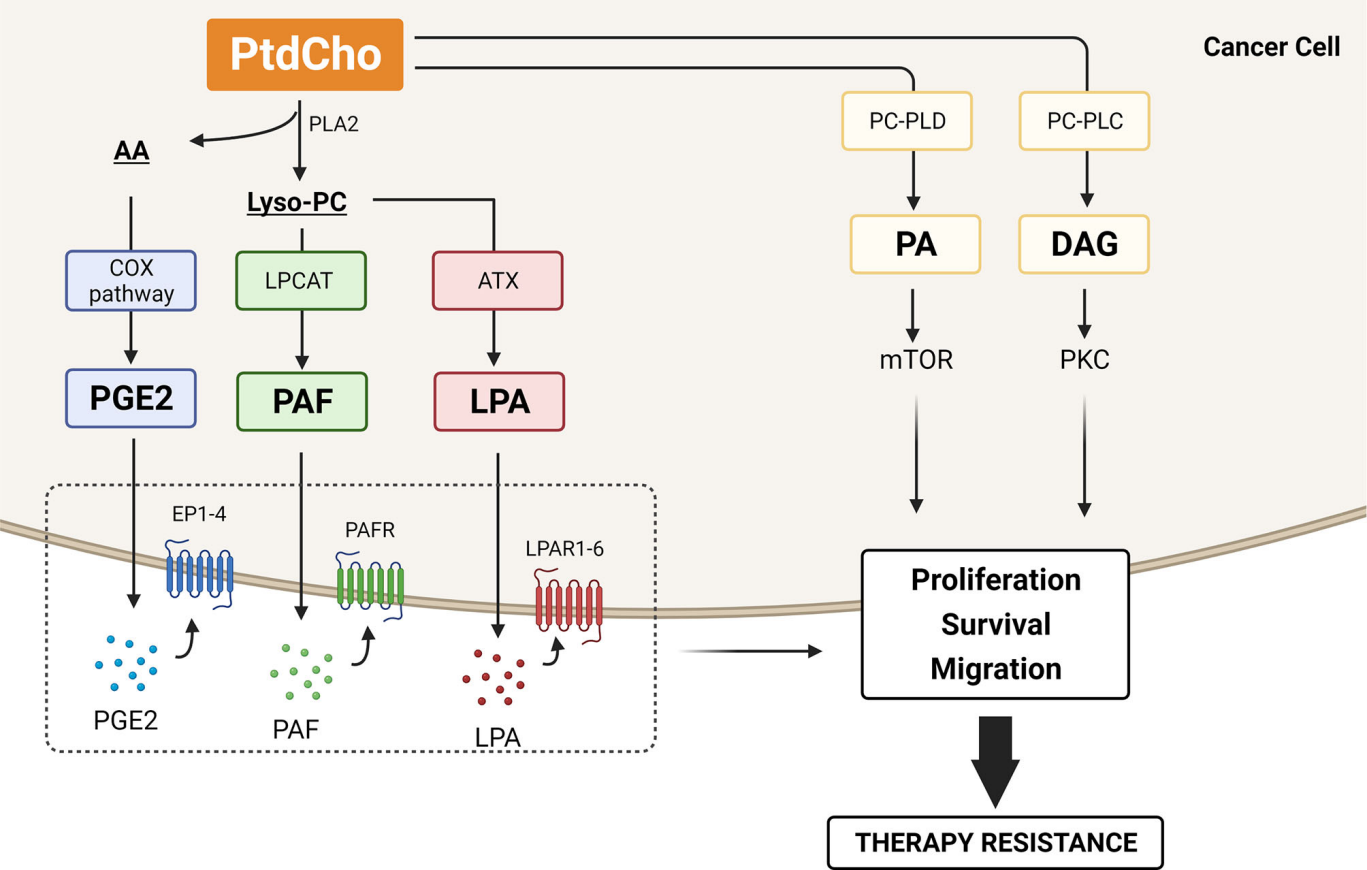
Lipid mediators generated by phosphatidylcholine (PtdCho) turnover that contribute to therapy resistance. PtdCho is hydrolyzed by phospholipase A2 (PLA_2_), resulting in the production of lysophosphatidylcholine (lyso-PC) and arachidonic acid (AA). The COX2 enzymes catalyze the conversion of AA to prostaglandin E2 (PGE2), and lyso-PC acetyltransferases (LPCATs) convert lyso-PC into platelet-activating factor (PAF). Alternatively, lyso-PC can be hydrolyzed by autotaxin (ATX) and generate lysophosphatidic acid (LPA). These three lipid mediators, PGE2, PAF, and LPA, are secreted and bind to their cognate receptors EP1-4, PAFR, and LPAR1-6, respectively, promoting cancer cell proliferation, survival, and migration. PtdCho is also hydrolyzed by phosphatidylcholine phospholipase C (PC-PLC) and D (PC-PLD), generating diacylglycerol (DAG) and phosphatidic acid (PA). DAG activates the protein kinase C (PKC) pathway, and PA is crucial for mTOR activity, promoting cancer cell proliferation and survival. All these catabolic products of PtdCho have been involved in therapy resistance. Created with BioRender.com.

PtdCho turnover (catabolism) is mediated by phospholipases (A2, C, and D), generating both choline-containing phospholipids (e.g., PhoC, GPC, and choline), that can be reutilized for PtdCho biosynthesis and lipid mediators that regulate multiple protumoral signaling pathways. The list of dysregulated bioactive lipids that have been shown to contribute to tumor biology includes AA, eicosanoids, DAG, PA, lysophosphatidic acid (LPA), platelet-activating factor (PAF), ceramide, sphingosine, and other lysosphingolipids (120). This list continues to grow, and here we highlight PtdCho-derived lipid mediators emerging as lipid second messengers involved in resistance to therapy (**Figure 4**).

More than 50% of PtdCho synthesized in the Kennedy pathway is remodeled through the Lands cycle (121). In this remodeling pathway, PtdCho is hydrolyzed by phospholipase A2 (PLA_2_), resulting in the production of lysophosphatidylcholine (lyso-PC) and AA. Once released, AA participates in the biosynthesis of eicosanoids such as prostaglandins and leukotrienes. Notably, AA is metabolized through the enzyme COX-2 into the terminal product PGE2. Elevated levels of COX-2 and PGE2 are frequently observed in many cancers and are associated with cancer initiation, progression, and resistance to therapy. Mechanistically, the activated COX-2/PGE2 pathway leads to therapy resistance mainly through affecting the TME by inducing epithelial–mesenchymal transition (EMT), suppressing anticancer immunity, and regulating cancer stem cell (CSC) homeostasis (122). Several reports with diverse cancer cell lines have shown that EMT is promoted by COX-2-induced PGE2 production, of which inhibition reverts this phenomenon (123–125). Moreover, EMT promoted by COX-2/PGE2 axis confers resistance to epidermal growth factor receptor (EGFR) tyrosine kinase inhibitor erlotinib (126).

Under physiological conditions, another PtdCho-derived lipid mediator generated from PLA2 activity, lyso-PC, is rapidly metabolized or reacylated to avoid the cytolytic induction caused by high intracellular concentrations due to its amphipathic property. The reacylation of lyso-PC is performed by lysophosphatidylcholine acyltransferases (LPCATs) by adding fatty acids at the sn-2 position to yield PtdCho, which rapidly gets recycled by the Lands cycle. These cycles of deacylation and reacylation of PtdCho modify the fatty acid composition of the phospholipids *de novo* generated in the Kennedy pathway and produce membrane asymmetry and diversity. Intracellular Lyso-PC concentration is also regulated by its hydrolysis through the enzymatic activity of autotaxin (ATX), an enzyme with lysophospholipase D activity, that generates LPA. Several cancers exhibit the activity of ATX enhanced and consequent increased levels of LPA (127) that is associated with cancer development and poor prognosis (128). Several reports have exploited the ATX–LPA signaling on cancer cell protection against chemotherapy and radiotherapy. Minami et al. (129) showed that LPA signaling *via* LPA receptor (LPAR5) regulates the resistance to cisplatin and dacarbazine in a melanoma cell line (129). Additionally, ATX–LPA signaling was reported to protect colon cancer cells from cisplatin and 5-fluorouracil-induced apoptosis (130) and to decrease cisplatin cytotoxic effect in human ovarian cancer cells (131). Inhibition of ATX activity reverts the protective effect of LPA on Taxol-induced apoptosis in breast cancer cells (132). The mechanism involved in ATX–LPA axis attenuation of chemotherapy-induced cell death includes the activation of PI3K–Akt survival pathway (132) and stabilization of nuclear factor E2-related factor 2 (Nrf2) transcription factor. Nrf2 increases the transcription of multidrug-resistant transporters and antioxidant genes, counteracting the chemotherapy-induced oxidative damage (133). Similarly, ATX inhibition enhances the radiotherapy-induced apoptosis in breast cancer cells (134) and attenuates radiation-induced survival, invasion, and angiogenesis in glioblastoma cells (135). LPA-mediated therapy resistance could also be attributed to its role in regulating tumor-associated macrophage (TAM) formation and tumor immunity (136–138).

In addition, to convert lyso-PC into PtdCho, LPCAT enzymes reacetylate lyso-PC and generate another lipid that is a potent cellular mediator, platelet-activating factor (PAF). Four enzymes (LPCAT1–LPCAT4) constitute the LPCAT family and, despite all LPCAT members being involved in lyso-PC conversion into PtdCho, only LPCAT1 and LPCAT2 are known to play an important role in PAF production (139, 140). We have demonstrated *in vitro* that the simultaneous silencing of all four LPCAT transcripts by modular nucleic acid nanoparticles resulted in lyso-PC (lyso-PAF 16:0 and 18:0) accumulation and enhanced the radiation cytotoxic effect in melanoma cells. We suggest that interfering in LPCAT-mediated signaling disturbs the generation of lyso-PAF, and PAF and contributes to cancer cell sensitization (141). PAF generally refers to alkyl-PAF, the most active form of PAF; however, abundant amounts of an acyl analog of PAF (acyl-PAF) is concomitantly generated with the alkyl PAF species. For a long time, acyl-PAF was considered an inactive PAF analog (142). Intriguingly, Chaithra et al. (143) have demonstrated *in vitro* and *in vivo* that acyl-PAF dampens PAF-R signaling and suppresses the action of alkyl-PAF (143). Accordingly, besides exerting their actions through a single PAF receptor (PAF-R), this pair of lipid mediators has opposite effects as inflammatory set-point modulators. The acyl-PAF has been neglected in PAF biology studies in the oncology field, and it is crucial to address the complex interplay between PAFR, alkyl-PAF, acyl-PAF, and their common catabolic enzyme PAF acetylhydrolase (PAF-AH) to unravel the role of PAF/PAFR signaling pathway. PAF is implicated in cancer progression by triggering inflammation and promoting proliferation, survival, metastasis, angiogenesis, and immune-suppressive responses (144). Elevated levels of PAF or increased PAF-R expression was observed in response to various stimuli, including therapeutic agents (145). As outlined, cisplatin increases PAF-R expression, and its inhibition by a PAF-R antagonist resulted in the chemosensitization of melanoma (146) and ovarian (147) cancer cells *in vitro* and *in vivo*. Additionally, PAF is generated following the treatment of B16F10 melanoma cells with chemotherapic agents such as etoposide, cisplatin, and melphalan. Importantly, elevated levels of PAF and oxidized lipids with PAF-R agonist activity were detected after the treatment with these drugs as a result of their common ability to induce ROS (148). Similar results were obtained after exposure of melanoma and cervical cancer cells to irradiation (149, 150). Interesting, in a murine melanoma model with a dual injection of B16F10 cells, treatment of one tumor with irradiation or chemotherapy augmented the growth of the untreated tumor in a PAF-R-dependent manner (150) (148). This evidence offers important insight into the systemic role of PAF and PAF-R agonists on negative regulation of therapy efficacy. In this sense, increased activation of PAF–PAFR axis impacts chemo/radioresistance through inducing immunosuppression by modulating regulatory T cells (Tregs) in a COX-2–dependent process (148). These results have driven the investigation of PAF/PAF-R axis in the tumor repopulation phenomenon.

The most prominent consequence of anticancer therapy is a massive induction of cell death frequently associated with a residual number of surviving tumor cells with the capacity to repopulate the tumor. The molecular mechanism involved in tumor repopulation has been investigated, and in 2011, Huang et al. (151) showed that in radiotherapy-induced apoptotic cancer cells, activated caspase-3 activates cPLA-2 and results in increased levels of PGE2, which as mentioned above can trigger protumoral signaling pathways and stimulate the growth of surviving tumor cells culminating in tumor repopulation (151). Interestingly, compelling evidence has indicated that PAF is at least partially responsible for this mitogenic effect of dying cells. Bachi et al. (152) reported that co-injection of apoptotic cells and a subtumorigenic dose of melanoma cells promote the tumor growth, and this phenomenon was inhibited by PAF-R antagonists (152). The following study showed that irradiated TC-1 cells promote the *in vitro* proliferation of TC-1 viable cells that was diminished by PAF-R antagonist treatment. In the same study, in an *in vivo* repopulation assay with a model of a human carcinoma cell line expressing (KBP) or not (KBM) PAF-R, the co-injection of live KBP cells and irradiated-induced dying KBM cells resulted in faster tumor growth compared with co-injection of a mixture of live and irradiated KBM cells (153).

Another catabolic route of PtdCho is mediated by PC-PLC enzyme that hydrolyzes PtdCho into PCho and DAG. The latter is probably the best-studied second messenger in cancer biology. It has been shown that the transformation of cells with oncogenes, such as *ras*, results in a prolonged and persistent elevation in DAG levels. Moreover, DAG activates the protein kinase C (PKC) pathway that is involved in several protumoral pathways, including cell cycle progression, tumorigenesis, and metastatic dissemination (154). Another lipid mediator, PA, is generated from the PtdCho hydrolysis mediated by PC-PLD. The mammalian target of rapamycin (mTOR) was reported as the main target of PA in cancer cells (155). The stability and activity of mTOR complexes depend on interaction with PA and result in signals for cancer cell survival (156). PA interacts with mTOR in a manner that is competitive with the mTOR inhibitor rapamycin, and as a consequence, elevated PC-PLD activity, frequently observed in tumors, confers rapamycin resistance (157). Upregulation of PLD2 was observed in multidrug-resistant colon and breast cancer cells, suggesting that PC-PLD could provide a survival signal involved in therapy resistance (158).

In line with the experimental observations mentioned above, it is not unreasonable to assume that cancer cells exhibit a prominent PtdCho degradation. The fact that PtdCho degradative enzyme activity (46, 159, 160) and PtdCho-derived mediator levels are frequently found elevated in tumors (161), cancer cells with increased PtdCho degradation hijack PtdCho-derived lipid mediators to favor tumor progression and enhance therapy resistance.

### Phosphatidylcholine-Coated Lipid Droplets Confer Resistance to Therapy

Lipid droplets (LDs) are predominantly formed by triacylglycerol (TAG) and PtdCho. In the last years, the role of these organelles in cancer has been well recognized, and more recently, some groups have made an effort to understand their role in tumor resistance. LD formation and accumulation were found in some drug-resistant cell lines, raising the possibility that these organelles might confer resistance to therapy (162–165). The association of choline metabolism and LD was demonstrated by the presence of active LPCAT1 and LPCAT2 in LDs by Moessinger et al. in 2011 (166). Recently, Cotte et al. (167) demonstrated that 5-fluorouracil and oxaliplatin-induced lipid droplet formation in colorectal cancer cell lines was supported by the enzyme LPCAT2 (167). Moreover, it was observed that LPCAT2-dependent lipid droplets conferred resistance to chemotherapy in these cells, and this effect could be reversed by inhibition of LD biogenesis, indicating the potential of LPCAT2 as a target to increase chemotherapy efficacy.

### Choline Metabolites Can Modulate DNA Methylation and DNA Repair

Some studies conducted in normal cells showed the relationship between choline and DNA. Due to the presence of three methyl groups in the nitrogen atom, choline can donate these methyl groups to the formation of S-adenosylmethionine (SAM) that is the main methyl donor for the epigenetic alteration in DNA and histones (168, 169). In 2004, Niculescu et al. (170) observed an increase in cyclin-dependent kinase inhibitor 3 (CDKN3) levels in choline-deficient neuroblastoma cells due to its hypomethylation leading to a reduction in proliferation, revealing that choline can interfere with tumorigenesis as a modulator of DNA methylation (170). A few years later, this finding was corroborated by studies using rodent models that showed that diets low in choline led to an increase in spontaneous hepatocarcinoma (revised in 171). Additionally, Kovacheva et al. demonstrated that choline deficiency was responsible for DNA methyltransferase 1 (DNMT1) overexpression due to its hypomethylation, which led to a global DNA hypermethylation in rats (172). These studies suggest that choline might contribute to methyl metabolism and DNA methylation and gene regulation in carcinogenesis and tumor progression.

Furthermore, it is known that the effect of cytotoxic therapy is mainly dependent on nuclear DNA damage extension and the DNA repair capacity of tumor cells to remove these lesions, and choline metabolism can interfere in this process. In 2007, Mori et al. (173) observed that under ChoK knockdown, the death ratio of 5-fluorouracil-treated breast cancer cells increased and, at the molecular levels, this effect was accompanied by a decrease in the expression of some DNA repair-related genes such as RAD23 that is known to participate in the nucleotide excision repair pathway (173). Using a rodent model to study carcinogenesis, choline deficiency was found to be correlated with the silencing of some tumor suppressor genes including the DNA repair genes *BRCA1* and *hMLH1*, indicating that this metabolite also modulates DNA stability (174).

### Phosphatidylcholine Metabolic Enzymes and Receptor Tyrosine Kinase Activation

In the last years, genome sequencing from tumor cells led to the identification of oncogenic mutations that are responsible for tumor cell survival and growth. Some of these mutations were found to be druggable, and blockade of the signaling pathways governed by them had improved cancer treatment in these cases. A noticeable example is the receptor tyrosine kinases (RTKs) that are often constitutively activated in different tumor types. Interestingly, some choline metabolites seem to participate in some of these oncogenic pathways controlled by RTKs. A possible correlation between RTK and choline metabolism was demonstrated by Pisanu et al. (175). The authors verified an increase in PC-PLC activity and PCho content in Human epidermal growth factor receptor 2, HER2--overexpressing ovarian cancer cells. Previously, Paris et al. (176) showed PC-PLC accumulation in the plasma membrane of HER2-overexpressing breast tumor cells (176). PC-PLC inhibition caused a downregulation in HER2 levels due to HER2 internalization that impaired its return to the cell membrane and the activation of HER2 signaling pathways. Moreover, the authors demonstrated that PC-PLC is physically associated with both HER2 and EGFR, and blockade of PC-PLC was able to reduce cell proliferation even in trastuzumab-resistant cells. These results provide evidence that PC-PLC is a promising target to counteract the oncogenic effect of HER2 amplification mainly in breast and ovary malignancies. Concerning the other molecules from choline metabolism, in 2012, it was shown that EGFR interacts with choline kinase α2 (ChoKα2) (177). The authors observed that c-Src-dependent phosphorylation sites of CHKA2 are necessary for EGF-dependent cell growth, suggesting that ChoKα may be an effective target for the treatment of tumors that overexpress EGFR and c-Src. In prostate cancer, the enzyme ChoKα was proposed to be a chaperone for androgen receptor, since its transcriptional activity was dependent on ChoKα. The inhibition of these choline kinases caused a decrease in cell proliferation *in vitro*, tumor growth, and metastasis *in vivo*, demonstrating its potential as a target for prostate cancer treatment (178). A recent work by Lin et al. (179) also described the association between ChoKα and EGFR in hepatocarcinoma (179). The authors observed that the pro-metastatic effect of ChoKα is mediated by its binding to EGFR, promoting its dimerization and AKT activation. Additionally, ChoKα overexpression promoted resistance to EGFR-targeted drugs both *in vitro* and *in vivo*, and the dual inhibition of ChoKα/mammalian target of rapamycin complex 2, mTORC2 might overcome the resistance to EGFR-targeted therapy in these tumors.

Still, under this context, PAF metabolite also interacts with EGFR in cancer cells. In ovarian tumor cells, PAF increased EGFR phosphorylation *via* PLCβ, intracellular Ca2+, Src, and the ADAM-mediated release of EGFR ligand HB-EGF, showing the interaction between PAFR and EGFR signaling pathways (147). More recently, one of the enzymes responsible for PAF production, LPCAT1, was also shown to be required for EGFR signaling. In GBM cells, EGFRvIII altered cell lipid composition through LPCAT1 that is, in turn, upregulated by EGFR. Knockdown of LPCAT1 was able to reduce tumor growth *in vivo*, indicating that targeting LPCAT1 can be a promising strategy to treat or reduce tumor recurrence in amplified EGFRvIII GBMs (180). These studies demonstrate the potentially actionable role of the choline-related enzyme in cancer treatment.

### Phosphatidylcholine Contributes to Autophagy-Induced Drug Resistance

Autophagy is a catabolic mechanism that plays an important role in the lysosomal degradation of protein aggregates, macromolecules, and damages organelles to recycle cellular components and sustain cell metabolism. This dynamic cellular self-digestion has a dual role in cancer cells, acting as a tumor suppressor or tumor promoter, depending on cancer type and stage. Cancer cells display activation of diverse processes to overcome stress, among them, autophagy. This program provides metabolic needs and helps cancer cells to sustain tumor viability and promote drug resistance (181, 182). While autophagy has been plenty studied, the role of lipids in this process is in its early stages, in part, due to the technical challenge of working with lipids. Phospholipids derived from the Kennedy pathway play an important role in the first phases of autophagy. PtdEth acts as an anchor of the microtubule-associated light protein light chain 3 (LC3), essential to cargo selection and autophagosome biogenesis (183). Recently, choline phospholipids (ChoPL), composed of phosphatidylcholines, sphingomyelin, and lysophosphatidylcholines, were reported in the autophagosome assembly. In this study, Andrejeva et al. (184) demonstrated that autophagy induced by anticancer drugs, followed by the incorporation of ^13^C-labeled choline, resulted in a high *de novo* synthesis of ChoPL in cancer cells. Moreover, to investigate the mechanism responsible for this process, they used MT58 cells that hold a temperature-sensitive mutation in the rate-limiting enzyme of PtdCho synthesis, CTP: phosphocholine cytidylyltransferase α1 (CCTα1). They showed that the loss of CCTα1 activity revokes autophagy and impairs cells to sustain autophagosome formation for extended periods of autophagy (184). By this, novel studies have also shown the importance of a second human CTP: phosphocholine cytidylyltransferase, CCTβ3 enzyme. In short-term starved cells, CCTβ3 is recruited to the autophagosome membrane to activate PtdCho synthesis and induce omegasome expansion. Despite that, CCTβ3 did not cause a meaningful upregulation of autophagy. However, opposite effects were observed in cells submitted to long periods of starvation, indicating that CCTβ3 is critical in the PtdCho synthesis to sustain prolonged autophagy (185). Additionally, the induction of autophagy in CCTβ-null cancer cells was significantly suppressed, and such effect was reversed by rescued expression of CCTβ3. Interestingly, the re-expression of CCTβ3 increased cell survival after starvation, indicating the relevance of PtdCho metabolism to autophagy activation and the subsequent impact in survival and resistance to therapy of cancer cells. In this sense, it was demonstrated that treatment with ChoK inhibitors, as B-3D and EB-3P, in liver cancer cells caused the reduction of autophagy components and induced apoptosis (186). Thus, it is likely that elevated levels of choline phospholipids observed in cancer cholinic phenotype may sustain drug-induced cytoprotective autophagy, which favors therapy resistance.

## Modulation of the Immune Microenvironment by Phosphatidylcholine-Derived Lipid Mediators

**Figure 5:**
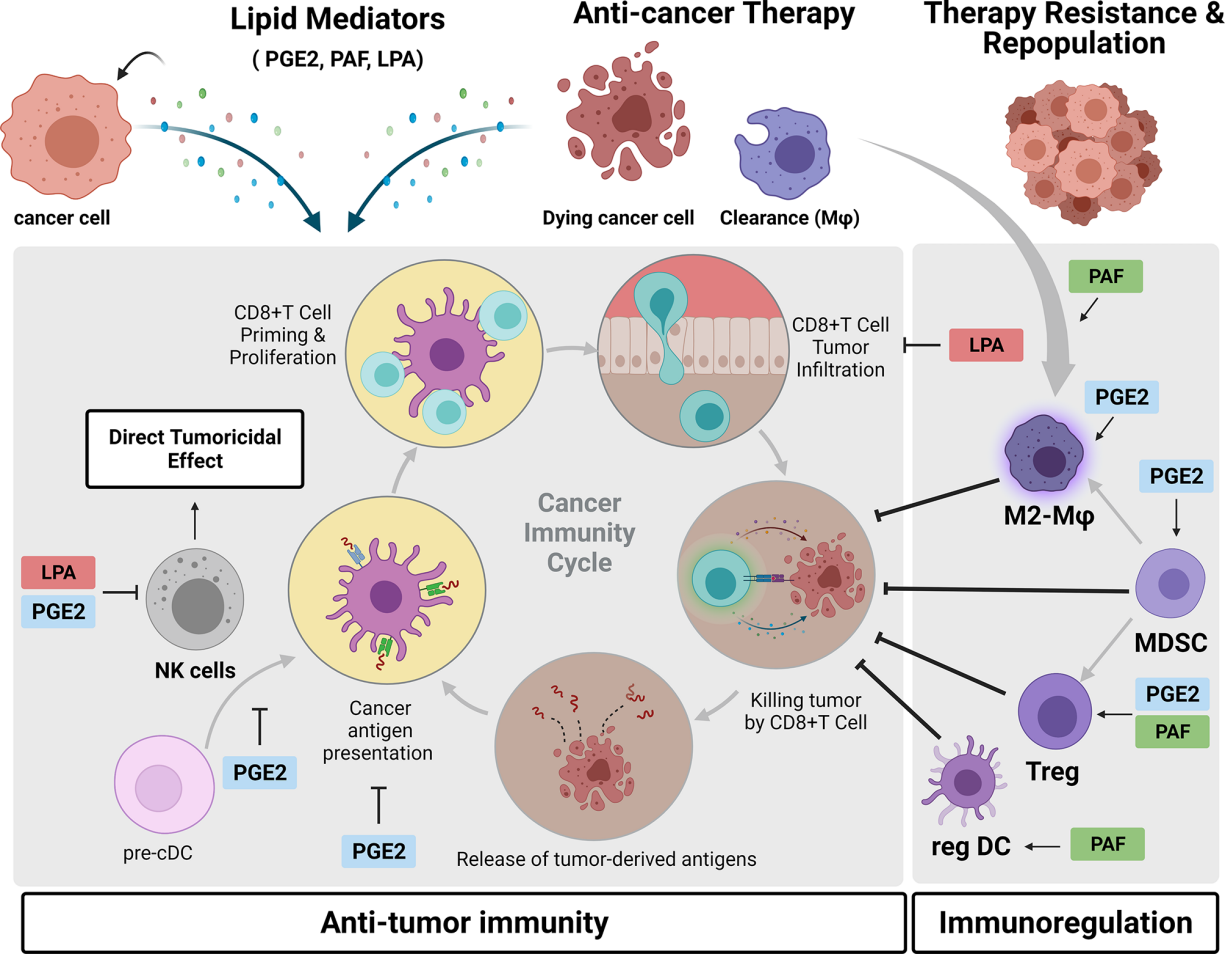
Crosstalk between cancer cells and the immune microenvironment mediated by phosphatidylcholine (PtdCho)-derived lipid mediators. During tumor progression and in response to anticancer treatments, cancer cells generate PtdCho-derived lipid mediators, such as prostaglandin E2 (PGE2), platelet-activating factor (PAF), and LPA. Once released in the tumor microenvironment, they bind to their cognate receptors present in diverse immune cells, inhibiting the antitumor immunity and promoting immunoregulation. These lipid mediators exert a complex interplay between tumor and immune cells that contributes to therapy resistance and tumor repopulation. Created with BioRender.com.

The studies that characterized the aberrant choline metabolism in cancer cells were based on two-dimensional (2D) tissue culture models. These reductionist models fail to reflect the complexities of TMEs that can influence cancer metabolic pathways. In line with this, Mori et al. (187) identified differences in Cho metabolites loads, especially PC and tCho, between cancer cells maintained in 2D monolayer culture and the corresponding tumor xenografts (187). This study reveals the importance of the TME in modulating choline metabolism. As mentioned above, TME conditions such as hypoxic, acidic, and areas of cell death can modulate Cho metabolic pathways. Additionally, altered tumoral PtdCho metabolism can mediate the interaction of cancer cells with TME components, such as immune cells, regulating the immune responses. Nishiyama-Naruke and Curi (188) found that PtdCho is incorporated by macrophages at higher rates than lymphocytes; afterward, it is secreted and transferred to these latest cells, promoting an antiproliferative effect (188). Lyso-PC, a class of lipids derived from the cleavage of PtdCho, can be recognized in the context of CD1d by a subpopulation of human T lymphocytes, called natural killer T (NKT) cells (189). Fox et al. (189) identified LPC as a self-antigen responsible for the activation of human NKT cells, specifically the subgroup known as invariant NKT (iNKT). In a murine context, PtdCho was determined to be in complex with murine CD1d by the crystal structure study; however, this last study did not address the activation of murine NKT by CD1d-mediated presentation of PtdCho (190). There is little information about the role of NKT cells in the TME compared to NK cells. However, differences in the distributions and phenotypic and metabolic profiles of NK vs. NKT cells have been observed in breast cancer and melanoma progression (191). Liu et al. (191) have demonstrated that NKT cells are exhausted in advanced cancers, contributing to the suppressive TME. A major contributor to tumor progression and the main obstacle for successful tumor immunotherapy is the suppression or dysfunction of immune cells, and PtdCho-derived lipid mediators play a role as an intercellular signal during tumor immune responses, promoting regulatory functions of diverse immune cells (**Figure 5**). It is worth noting that lipid mediators exert their biological effects by binding to cognate receptors (192), which can be expressed in cancer cells and stromal cells. Considering that several PtdCho-derived lipid mediators can be generated by both cells, mainly immune cells, we will further extend our discussion on the impact of PtdCho-derived mediators on therapy resistance by exploiting the role of these lipid mediators in the crosstalk between cancer and immune cells.

There is extensive literature describing that COX-2/PGE2 axis triggers tumor immune evasion in multiple ways leading to disease progression and therapy resistance (193). Tumor-derived PGE2 promotes the activity of the main immunosuppressive cells in the TME, such as myeloid-derived suppressor cells (MDSCs) (194, 195), M2-like macrophages (196, 197), and Tregs (198, 199). PGE2 is also reported to promote inactivation of antitumor immune response by directly impairing NK activity (200) and also inhibition of NK–dendritic cell (DC) crosstalk, which is crucial for DC recruitment into the tumor (201). Modulatory effects of PGE2 on DC have also been described and show that PGE2-primed DC has increased production of the anti-inflammatory cytokine interleukin 10 (IL-10) and decreased antigen-presenting cell, APC activity, inducing the development of a tolerogenic subset of DCs (202) (203). Recently, evidence shows that tumor-derived PGE2 promoted programmed cell death protein ligand 1 (PD-L1) expression on tumor-infiltrating myeloid cells and, therefore, plays an important role in tumor escape from anti-PD-L1 immunotherapies (204). Altogether, these effects of PGE2 drives tumor to a non-T cell-inflamed status, a crucial refractory condition to cancer immunotherapies. Indeed, COX-2 inhibition and consequently diminished levels of PGE2 reduce the infiltration of MDSC in the TME along with a lymphocyte-mediated antitumor response (194, 195). A study with viral vectors engineered to target PGE2 demonstrated that this viral therapy was able to reduce intratumoral MDSC and sensitize tumors to anti-PD-1 treatment (205). Several *in vivo* studies have demonstrated an antitumoral effect of selective inhibitors of the prostaglandin E receptor 4 (EP4), one of four PGE2 receptors. These EP4 antagonists suppressed tumor growth by NK cell function reactivation and DC repopulation together with an increase in CD8^+^ T-cell frequency while decreasing M2-like macrophage polarization (206). Similarly, the effect of switching from an immunosupressive response to antitumor response was observed with inhibition of EP2 receptor (207).

PAF is another PtdCho-derived lipid mediator with immunoregulatory activity. The main idea is that therapy-induced PAF/PAFR axis activation could result in systemic immunosuppression that reduces therapy efficacy. In line with this, evidence shows that PAFR is essential in the clearance of apoptotic cells and induces a regulatory phenotype of macrophages (208, 209). Another piece of evidence also shows that implanted tumors in mice that do not express PAFR (PAFR KO) exhibited higher infiltration of M1-like (CD11c^+^) and lower M2-like (CD206^+^) macrophages (153). Similarly, PAFR activation in DC has been shown to induce a regulatory phenotype of these cells characterized by an increase in IL-10 and PGE2 production, which was blocked by PAFR antagonists (210). These data suggest that either the phagocytosis of therapy-induced apoptotic cells or the binding of therapy-generated PAF to macrophages or DCs results in an M2-like phenotype (TAMs) and regulatory DCs, respectively, in the TME. Moreover, Tregs and MDSCs participate in the PAF-mediated increased growth of B16F10 melanoma tumors. Sahu et al. (211) reported that UVB-generated PAFR agonists potentiate the tumor growth of B16F10 cells, a phenomenon that was reversed by depletion of Tregs *via* anti-CD25 neutralizing antibodies (211).

Recently, emerging evidence of LPA has addressed the role of this lipid mediator in the crosstalk between cancer cells and TME cells (137). It has been demonstrated that LPA negatively modulates antitumor immunity *via* suppressing NK activity (212), inhibiting CD8^+^ T-cell infiltration and activity (138, 213). Interestingly, it was reported that a predominant source of LPA production in the TME is derived from a consecutive action by platelet-activating factor acetylhydrolase (PAF-AH) and ATX in TAM (214). In parallel, LPA has been reported to mediate TAM formation by activating the PI3K/AKT/mTOR signaling pathway through LPAR receptor activation, describing an LPA vicious cycle that contributes to malignant features of ovarian cancer (136). Although emerging data on immunomodulatory actions of LPA in the context of cancer immunity have been reported, there are open questions of how LPA regulates other TME cells, including Tregs, MDSCs, TAMs, and CD4^+^ T cells.

Taken together, all these reports demonstrate that a variety of PtdCho-derived lipid mediators support an immunosuppressive TME that compromises the therapeutic efficacy of anticancer treatments. As outlined, chemo and radiotherapy-induced tumor cell debris generates lipid mediators, in particular PGE2 (151, 215) and PAF (153), that create a protumorigenic TME, favoring the growth of residual surviving cancer cells. This has unveiled an insight into the mechanism behind the tumoral repopulation process and has driven the investigation of whether stimulating the clearance of therapy-generated debris could mitigate this phenomenon. Emerging evidence indicates that the chronic inflammation associated with tumor growth is promoted by a failure in the resolution of inflammation (216, 217), a process coordinated by specialized pro-resolving mediators (SPMs), such as resolvins, a family of endogenous lipid mediators that counteract pro-inflammatory cytokines and increase the macrophage-mediated clearance of cell debris. Sulciner et al. (218) reported that resolvins (RvD1, RvD2, or RvE1) inhibit therapy-generated debris stimulation of tumor growth (219). Interestingly, increased levels of resolvin (RevE1) were detected in the plasma of healthy individuals after administration of aspirin (219). Moreover, low-dose aspirin inhibited experimental tumor growth and metastasis by triggering SPM generation, identifying a resolving receptor-dependent mechanism of aspirin chemopreventive activity (220). These findings have unveiled an exciting pro-resolving strategy to enhance the effectiveness of current cancer therapies and prevent a recurrence.

### Extracellular Vesicles as a Communication Route of Phosphatidylcholine Metabolites Between Cancer Cells and Tumor Microenvironment Cells

The modification in lipid cellular composition observed in cancer cells has also been noticed in the extracellular vesicles (EVs) secreted by them. EVs are nanostructures delimited by a lipid bilayer that carry a range of biologically active macromolecules like RNAs, DNA, protein, lipids, and cytokines. These spherical structures can bind to the plasma membrane or be engulfed by recipient cells, leading to a reprogramming that affects their functionality in the TME. Exosomes, a type of EVs that originated from multivesicular bodies, with bioactive lipids, such as PGE2α, PGE1, and PGE2, are secreted by macrophages and tumor cells into the TME (221).

sPLA2, cPLA2, iPLA2, COX-1, COX-2, AA, and PGE2 were already identified in tumor-derived EVs (204, 222–224). Concerning PGE2, EVs carrying this prostaglandin were shown to be associated with immune escape (204) and release of pro-inflammatory cytokines responsible for MDSC recruitment in breast cancer microenvironment (225, 226). In addition, the blockade of PGE2/EP4 signaling reduced the secretion of EVs by basal mammary epithelial stem cells while promoting the release of EVs and CSC-associated proteins from transformed mesenchymal breast cancer cells, modulating tumor progression. Although the relationship between choline metabolism and EVs is still an unexplored field, one might propose that lipid metabolism indeed affects the production and secretion of EVs, as well as interactions with the recipient cells, and more evidence showing the consequences of altered choline metabolism in tumor-derived EVs and their effects in the TME is a matter of time (227).

## Choline Metabolism and Cancer Diagnosis

The abnormal choline metabolism frequently described in cancer stimulated the development of strategies to evaluate this differential metabolic alteration in cancer diagnosing. One technique that has been used to quantify the metabolic profile of tumor tissues is a high-resolution magic angle spinning (HR-MAS) proton magnetic resonance spectroscopy (^1^H MRS). ^1^H MRS helps in the detection of increased choline expression and CHKa activity in cancer cells compared to those in non-tumoral cells, making it a potential biomarker to diagnose cancer and a strategy to follow treatment response (228). Although ^1^H MRS exhibits high sensitivity, the adoption of reliable tCho quantification in the clinics is challenging due to spatial localization errors and overlapping signals from PC, GPC, and Cho. On the contrary, the use of ^31^P-MRS spectra allows the individual detection of PC, GPC, and GPE metabolites but has lower sensitivity compared with that of ^1^H MRS (229, 230). Thus, the improvement and the combination of both techniques can be used to do cross-calibration and obtain more accurate results. While several studies use ^1^H MRS and ^31^P-MRS to aid in the diagnosis of different types of cancers (228, 231, 232), their use in the clinics is not yet broadly applied.

Another technique to detect increased choline metabolism is positron emission tomography/computed tomography (PET/CT) imaging with tracers. Along with the development of radiolabeled choline analogs, PET imaging, combining metabolic activity and anatomical structure (CT), has gained importance to visualize choline metabolism, providing more definitive diagnostic information (227). The main tracers available in the clinics and approved by the U.S. Food & Drug Administration (FDA) to use in PET imaging are [^11^C]-choline, [^18^F]-fluoroethylcholine, and [^18^F]-fluoromethylcholine. Still, there are no guidelines yet for image acquisition, and they are not widely available due to the high cost and the need for further development (233, 234). Currently, the combination of PET/magnetic resonance imaging (MRI) is being evaluated, since it could have complementary functions that provide more robust data (227).

## Alternatives to Specifically Target Phosphatidylcholine Metabolism to Treat Cancer

Based on the protumoral effects associated with aberrant choline activity in tumors, investigations have been conducted to target several components of choline metabolism. A well-explored drug target is the inhibition of ChoK activity. ChoK inhibitors or *ChoKα* gene silencing by RNA interference has been developed to target ChoK, the enzyme responsible for sustaining PCho biosynthesis (235). Interestingly, studies have shown that downregulation of *ChoKα* decreased epithelial ovarian cancer cell aggressiveness and increased drug sensitivity (117, 236). Moreover, in ovarian cancer cells, *ChoKα* impairment overcomes Tumor Necrosis Factor (TNF)-Related Apoptosis-Inducing Ligand (TRAIL) resistance (237). Similar results were also obtained with different pharmacological ChoK inhibitors as hemicholinium-3 (HC-3), a choline transport blocker that presents high toxicity *in vivo* (238) and chemically modified HC-3 structures, MN58b and RSM932A (also TCD-717). Further modification in MN58b and RSM932A produced novel inhibitors, such as EB-3D and EB-3P, respectively (235). These inhibitors exhibited anticancer activity and decreased cell proliferation in preclinical models (186, 239, 240). RSM932A inhibitor resulted in the most prominent *in vivo* antitumoral effect, retarding tumor growth in mouse xenograft without associated toxicity (240). RSM932A was the first inhibitor to enter a phase I clinical trial in patients with advanced solid tumors, and although this study has been ended, no data are available yet (186).

There are also other inhibitors targeting several components of the choline metabolism, such as PC-PLD1, PC-PLC, and choline transporters (**Table 1**). Recently, a novel strategy was designed to modify cancer cell membranes to prevent tumor proliferation. The investigators synthesized PtdCho-reversed choline phosphate lipid-modified with a PD-L1 antibody. Then, this structure was loaded in nanoparticles along with drugs to interact with melanoma cell membranes interfering in its functionality and rigidity, therefore reducing tumor growth and migration (247). Curiously, although inhibitors, drugs, and strategies to target PtdCho pathway have been generated, there is still no established molecule for use in the clinic, and resistance to ChoK inhibitor-induced antitumor effects has also been reported (248). This notion reinforces that it is necessary to investigate more selective and efficient inhibitors of the PtdCho pathway. To that end, it is crucial to clarify the association between local and systemic measurements of PtdCho and their metabolites. Systemic changes can be assessed by lipid quantification in cancer patient serum; however, *in vivo* measurements of these lipids in the TME is still a challenge. Considering that these lipids are susceptible to degradation or acetylation reactions, serum measurements do not necessarily correspond to TME levels. Thus, one of the most interesting remaining questions is how serum levels of PtdCho and their derivatives correlate with the actual concentration of these molecules within the local TME and their effects. Methodology improvement in the *in vivo* lipid measurement and strategies to specifically target lipid enzymatic synthesis in cancer cells will allow the study of PtdCho tumoral local effects and will be critical to determine the precise therapeutic window to effectively target this lipid pathway.

**Table 1:** Targeting PtdCho metabolism—current strategies for experimental cancer control and treatment.

Drugs/Inhibitors	Target	Anticancer effect	References
MN58b	choline kinase (ChoK)	-Synergism with Tumor Necrosis Factor (TNF)-Related Apoptosis-Inducing Ligand (TRAIL), inhibiting tumor growth in colorectal tumors *in vivo* -Growth arrest and apoptosis in brain tumor cells -Antiproliferative activity and synergistic effect with gemcitabine, 5-Fluorouracil (5-FU) in Pancreatic ductal adenocarcinoma (PDAC) cells	(241) (242)
RSM932A	ChoK	-Tumor growth inhibition and synergism with 5-FU in colorectal cancer model	(243)
EB-3D	ChoK	-Impaired proliferation, migration, and invasion as wells as induction of senescence of breast cancer cells *in vitro* and *in vivo*	(240)
EB-3P	ChoK	-Cell growth inhibition, mitochondrial alteration, and endoplasmic reticulum (ER) stress response	(186)
VU0359595/VU0285655-1	Phospholipases D1, 2	-Blockage of autophagic flux, promoting cancer cell death in glucose deprivation conditions -Reduction of cell survival and colony formation in prostate cancer cells	(244) (245)
FIPI	Phospholipases D1, 2	Inhibition of tumor growth and metastasis *in vivo*	(246)
D609	phosphatidylcholine-specific phospholipase C	-Induced loss of mesenchymal traits in metastatic breast cancer cells	(34)
Amb4269951/Amb4269675	choline-transporter-like protein 1 (CTL1)	-Inhibition of cell viability and increased caspase 3/7 activation in pancreatic cells. Inhibition of tumor growth (xenograft)	(26)

PtdCho, phosphatidylcholine.

## Conclusion

Abnormal choline metabolism drives cancer cell growth, survival, proliferation, and resistance to therapies in part due to the metabolism of PtdCho, which generates lipid mediators that in turn interfere with immune cell functions. These specific lipid mediators are also produced by immune cells and mediate complex crosstalk that results in immunoregulation and the development of therapy resistance. Controlling lipid metabolism represents a promising strategy for both the inhibition of therapy-induced tumor repopulation and the generation of a sustained antitumor immune response. The development of strategies toward cancer control and treatment through interference with PtdCho metabolism, however, relies on finding the right window of opportunity (when and for how long) for effective treatment.

## Author Contributions

All authors participated in the review conceptual design. RS, LA, and SB wrote, reviewed, and edited the article. RC revised and edited the article. All authors contributed to the article and approved the submitted version.

## Funding

This work was supported by grants CNPq 426714/2016-0 and 305700/2017-0 and FAPESP/SPRINT 17/50029-6.

## Conflict of Interest

The authors declare that the research was conducted in the absence of any commercial or financial relationships that could be construed as a potential conflict of interest.

## Publisher’s Note

All claims expressed in this article are solely those of the authors and do not necessarily represent those of their affiliated organizations, or those of the publisher, the editors and the reviewers. Any product that may be evaluated in this article, or claim that may be made by its manufacturer, is not guaranteed or endorsed by the publisher.
